# Overlapping and differential functions of ATF6α versus ATF6β in the mouse heart

**DOI:** 10.1038/s41598-019-39515-5

**Published:** 2019-02-14

**Authors:** Robert N. Correll, Kelly M. Grimes, Vikram Prasad, Jeffrey M. Lynch, Hadi Khalil, Jeffery D. Molkentin

**Affiliations:** 1 Department of Biological Sciences, University of Alabama, Tuscaloosa, Alabama 35487 USA; 2 Department of Pediatrics, University of Cincinnati, Cincinnati Children’s Hospital Medical Center, Cincinnati, Ohio 45229 USA; 3 Howard Hughes Medical Institute, Cincinnati, Ohio 45229 USA

## Abstract

Hemodynamic stress on the mammalian heart results in compensatory hypertrophy and activation of the unfolded protein response through activating transcription factor 6α (ATF6α) in cardiac myocytes, but the roles of ATF6α or the related transcription factor ATF6β in regulating this hypertrophic response are not well-understood. Here we examined the effects of loss of ATF6α or ATF6β on the cardiac response to pressure overload. Mice gene-deleted for *Atf6* or *Atf6b* were subjected to 2 weeks of transverse aortic constriction, and each showed a significant reduction in hypertrophy with reduced expression of endoplasmic reticulum (ER) stress-associated proteins compared with controls. However, with long-term pressure overload both *Atf6* and *Atf6b* null mice showed enhanced decompensation typified by increased heart weight, pulmonary edema and reduced function compared to control mice. Our subsequent studies using cardiac-specific transgenic mice expressing the transcriptionally active N-terminus of ATF6α or ATF6β revealed that these factors control overlapping gene expression networks that include numerous ER protein chaperones and ER associated degradation components. This work reveals previously unappreciated roles for ATF6α and ATF6β in regulating the pressure overload induced cardiac hypertrophic response and in controlling the expression of genes that condition the ER during hemodynamic stress.

## Introduction

Hemodynamic stress, such as that caused by chronic hypertension or aortic stenosis leads to activation of signaling pathways such as calcineurin/nuclear factor of activated T-cells and calcium-calmodulin-dependent protein kinase II that result in hypertrophy of the heart^1^. This growth initially serves as an adaptive response that allows for the maintenance of cardiac output but if the stress is prolonged the heart can decompensate leading to failure and death. It has been previously demonstrated that cardiac hypertrophy occurs simultaneously with activation of the unfolded protein response (UPR)^2,3^ in the endoplasmic reticulum (ER), a distinct set of signaling pathways designed to upregulate the protein folding and secretory capacity of cells during periods of stress^4^. The impetus for activation of UPR signaling in the hypertrophic heart is unclear, but is likely due to both stress-dependent dysregulation of the ER microenvironment required for proper protein folding as well as increased demand for total protein production in general.

UPR signaling is primarily initiated by three canonical ER resident effector proteins, protein kinase R (PKR)-like endoplasmic reticulum kinase (PERK), inositol-requiring enzyme 1 (IRE1), and activating transcription factor 6α (ATF6α)^4^. Accumulation of misfolded proteins results in trafficking of ATF6α (encoded by the *Atf6* gene) to the Golgi where it undergoes sequential cleavage by specific proteases, releasing an N-terminal portion that translocates to the nucleus where it activates expression of many ER protein chaperones, proteins involved in ER-associated protein degradation (ERAD) and other ER stress-inducible proteins^5,6^. Our previous work has demonstrated that ATF6α trafficking to the Golgi requires thrombospondin-4 (Thbs4) binding to the C-terminus^2^. Overexpression of Thbs4 drives activation of ATF6α even in the absence of ER stress, and transgenic mice with cardiac-restricted expression of Thbs4 are protected after myocardial infarction (MI)^2^. Indeed, ATF6α is broadly protective to the heart as shown with an *ex vivo* ischemia/reperfusion (I/R)^7^ model and in response to myocardial infarction (MI) injury *in vivo*^8^. More recently mice lacking the *Atf6* gene showed increased cardiac damage upon I/R injury^9^, although the role of the related gene *Atf6b* (encodes ATF6β protein) in the heart is less well understood.

While ATF6α signaling appears to play an important role in cellular protection following acute MI or I/R injury, less is known about its role in regulating hypertrophy and compensation^1^. However, previous results have shown that ATF6α and UPR signaling are activated after pressure overload hypertrophy and that mice lacking the *Thbs4* gene cannot activate ATF6α in response to pressure overload, which coincides with increased mortality in that model^2^.

Here we show that gene-deleted mice lacking either *Atf6* or *Atf6b* have significantly reduced hypertrophy after 2 weeks of pressure overload stimulation with reduced expression of some ER stress-associated proteins. Moreover, *Atf6* or *Atf6b* null mice, and *Atf6*^+/−^ *Atf6b*^+/−^ double heterozygous targeted mice each showed accelerated decompensation and heart failure after long-term (8 week) pressure overload stimulation. Microarray studies using cardiac-specific transgenic mice expressing the transcriptionally-active ATF6α or ATF6β N-terminus revealed partially-overlapping gene expression programs including ER protein chaperones and ERAD components. These data suggest that the increased demand on protein production and folding machinery associated with the hypertrophic response equally requires ATF6α and ATF6β signaling and we hypothesize that failure to activate these effectors compromises ER protein production resulting in heart failure.

## Results

**Figure 1:**
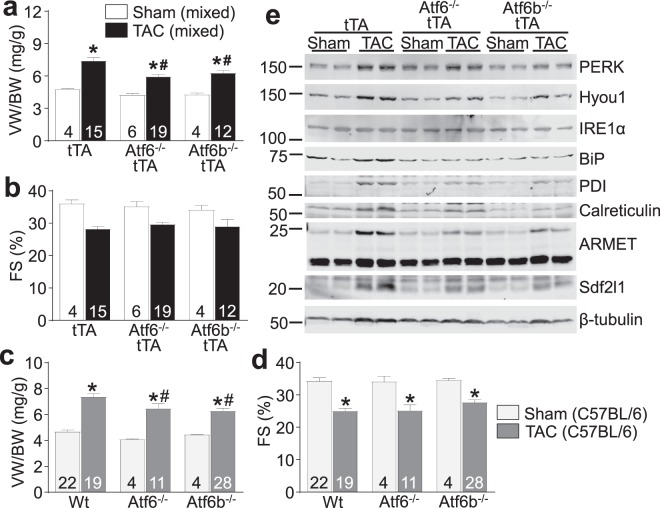
Mice lacking ATF6α or ATF6β protein have reduced hypertrophy after short-term pressure overload. (**a**) Gravimetric measurement of ventricle weight normalized to body weight (VW/BW) and (**b**) echocardiographic measurement of fractional shortening percentage (FS%) from *Atf6*^−/−^, *Atf6b*^−/−^, or control mice all also expressing the tTA transgene after 2 weeks of TAC or a sham surgery. (**c**) Gravimetric measurement of VW/BW and (**d**), echocardiographic measurement of FS% from *Atf6*^−/−^, *Atf6b*^−/−^, or control mice in a pure *C57BL/6* background without the tTA transgene after 2 weeks of TAC or a sham surgery. For each experiment, number of mice analyzed is given within the graph. *P < 0.05 versus sham of same genotype; ^#^P < 0.05 vs Wt TAC for TAC comparisons only (Newman-Keuls multiple comparisons test). (**e**) Immunoblots of ER stress-associated proteins from heart homogenates of Atf6^−/−^, Atf6b^−/−^, or control mice all also expressing tTA after 2 weeks of TAC.

We previously demonstrated that mice lacking *Thbs4*, which is required for proper ATF6α activation in the heart, showed increased mortality after transverse aortic constriction (TAC) surgery^2^. To examine the interplay between Thbs4 and ATF6α during pressure overload, we obtained gene-deleted mice lacking *Atf6* or *Atf6b*^10^ (ATF6β protein did not interact with Thbs4^2^) and crossed them with our previously-described cardiac-specific transgenic mice expressing Thbs4^2^ through an inducible tet transactivator (tTA) transgenic system. These mice were subjected to 2 weeks of pressure overload produced by TAC surgery, although our results showed that Thbs4 overexpression did not alter this TAC-induced hypertrophic response in either the *Atf6* or *Atf6b* null backgrounds (Fig. S1). Unexpectedly, we observed that eliminating the *Atf6* or *Atf6b* genes resulted in a significant reduction in cardiac hypertrophy (Figs 1a and S1a) but function was not affected over this relatively short 2 week time period (Fig. 1b). Although our previous studies show that the tTA transgene (expressed in the *Atf6*^−/−^ or *Atf6b*^−/−^ background) has no appreciable effect on cardiac structure-function, we repeated our entire study in a pure *C57BL/6* background that is more sensitive to TAC^11^, and without the Thbs4 or tTA control transgenes to eliminate these variables (Fig. 1c,d). Notably, wildtype (Wt) mice in the pure *C57BL/6* background demonstrated a significant decrease in fractional shortening after TAC, as opposed to those mice in the mixed *FVB/N* background (Fig. 1d), agreeing with previous experiments that suggest the *C57BL/6* strain is more sensitive to pressure overload^11^. These data again showed a similar significant reduction in cardiac hypertrophy in *Atf6*^−/−^ or *Atf6b*^−/−^ mice following 2 weeks of pressure overload hypertrophy without enhanced decompensation compared with Wt controls (Fig. 1c,d).

Examination of protein extracts from the hearts of mixed background mice after TAC revealed a trend towards increased expression of many ER stress-activated proteins and chaperones such as binding immunoglobulin protein (BiP), calreticulin, hypoxia up-regulated protein 1 (Hyou1), arginine-rich, mutated in early-stage tumors (ARMET), protein disulfide isomerase (PDI), stromal cell derived factor 2 like 1 (Sdf2l1), as previously described by us^2^, and gene-deleted mice lacking *Atf6* or *Atf6b* showed a trend towards reduced expression of some of these ER stress responsive proteins (Fig. 1e). In order to better quantify the reduction in ER stress protein expression after loss of *Atf6* or *Atf6b* (due to the small sample size and mixed background in Fig. 1e), we repeated our immunoblotting experiments using samples from the pure *C57BL/6* strain background after TAC (Fig. S2) and quantified them (Fig. S3). The results showed significant post-TAC inhibition of calreticulin expression with deletion of either *Atf6* or *Atf6b*, significant reductions in expression of PDI and ARMET with deletion of *Atf6b*, and a trend towards reduced expression of Hyou1 with deletion of *Atf6b*. However, qPCR analysis of mRNA levels for cardiac fetal gene program markers showed activation across these 3 groups of mice (Table S1).

**Figure 2:**
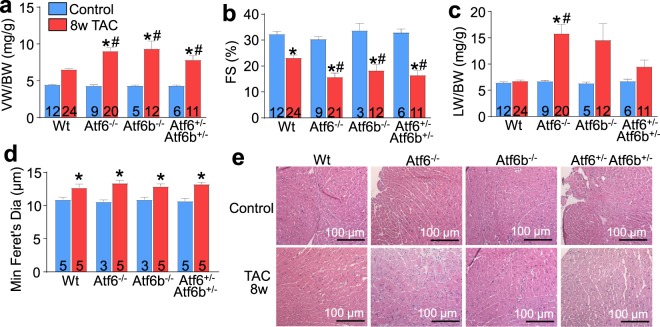
Mice lacking ATF6α or ATF6β protein, or double heterozygous mice have accelerated decompensation and failure after long-term pressure overload. (**a**) Gravimetric measurement of VW/BW (**b**), echocardiographic measurement of fractional shortening percentage (FS%) and (**c**), gravimetric measurements of lung weight normalized to body weight (LW/BW) from *Atf6*^−/−^, *Atf6b*^−/−^, *Atf6*^+/−^ *Atf6b*^+/−^, or control mice after 8 weeks of TAC. (**d**) Histological analysis of myocyte size using minimum Feret’s diameter from (**e**), H&E stained heart sections taken from *Atf6*^−/−^, *Atf6b*^−/−^, *Atf6*^+/−^ *Atf6b*^+/−^, or control mice after 8 weeks of TAC. Number of mice analysed is shown in the bars of each panel. The histology myocyte area measurements were taken from at least 4 separate sections from each of the mice shown in the panel. ^*^P < 0.05 versus control of same genotype; ^#^P < 0.05 vs Wt TAC for TAC comparisons only (Newman-Keuls multiple comparisons test).

We also hypothesized that the reduction in cardiac hypertrophy over 2 weeks due to a presumed alteration or reduction in ER protein production associated with the *Atf6* or *Atf6b* null backgrounds might render these hearts more susceptible to heart failure with chronic hypertrophic stimulation. Indeed, here we observed that after 8 weeks of TAC-induced pressure overload stimulation *Atf6*^−/−^ and *Atf6b*^−/−^ mice each showed greater heart weights, enhanced pulmonary edema and a more precipitous loss of function as assessed by echocardiography, compared with Wt controls (Fig. 2a–c). A limitation of this study is that the Wt TAC treatments did not show a significant increase in VW/BW ratio by one-way ANOVA. However, this is likely due to the Newman-Keuls post-hoc test we used, as an analysis using Student’s t-test showed that Wt TAC VW/BW ratio was significantly greater (p < 0.0001) than Wt control VW/BW ratio. We also attempted to generate *Aft6* x *Atf6b* double gene-deleted mice, or 3 of 4 allele combinatorial targeted mice, but all such mice were embryonic lethal, suggesting that these 2 genes play overlapping roles during development. However, we were able to generate double heterozygous mice (*Atf6*^+/−^ *Atf6b*^+/−^) and they showed the same increased propensity to heart failure as either single null line after 8 weeks of TAC (Fig. 2a–c). Such results suggest that the total level of “ATF6” protein is important in maintaining ER homeostasis in response to hypertrophic stress and that both genes appear to function in a redundant manner in the heart. Fibrotic remodeling was only significantly increased in the *Atf6*^−/−^ hearts after 8 weeks of TAC (Fig. S4a) and there was no difference in overall apoptosis rates as quantified by TUNEL in heart histological sections subjected to cardiac troponin T co-staining to enrich for cardiomyocyte scoring (Fig. S4b). Width of the myocytes as measured by minimum Feret’s diameter^12^ was increased after 8 weeks of TAC but it did not vary between genotypes (Fig. 2d,e). Given the heart failure phenotype and the significantly greater heart weights in the gene-targeted groups compared to the Wt controls, we hypothesize that the worsening phenotype with gene targeting of *Atf6* or *Atf6b* is due to greater myocyte lengthening since a modified measure of cross-sectional area was unchanged.

**Figure 3:**
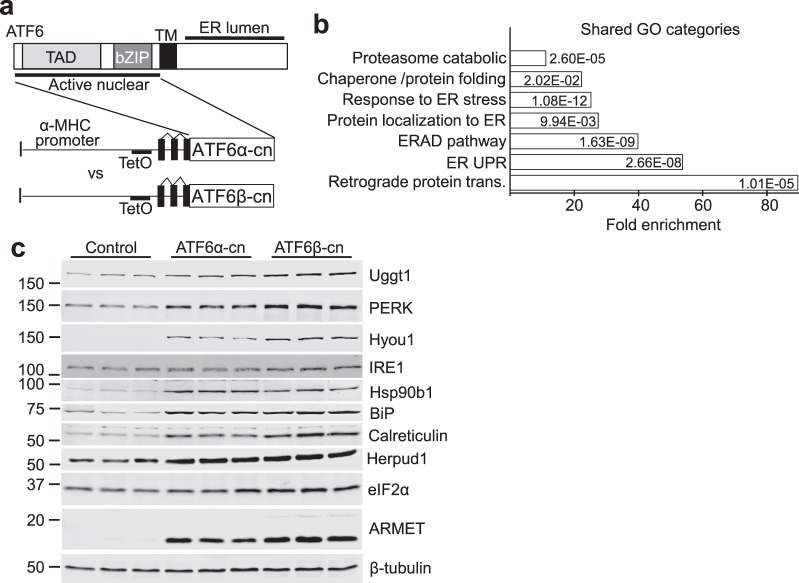
ATF6α and ATF6β control overlapping gene programs in the heart. (**a**) Schematic representation of the transgenes used to drive expression of ATF6α-cn or ATF6β-cn in the mouse heart. (**b**) Graph representing top significant GO categories regulated by both ATF6α-cn and ATF6β-cn along with average fold enrichment, through global gene array profiling (Student’s t-test). (**c**) Immunoblots of the indicated ER stress-associated proteins from heart homogenates of ATF6α-cn, ATF6β-cn, or control mice at 8 weeks of age (4 weeks off DOX).

We found it surprising that ATF6β, which was previously described as having only weak transcriptional activity compared with ATF6α^13–15^, was equally important in facilitating acute pressure overload-induced hypertrophy, inducing expression of ER stress-associated proteins and in maintaining cardiac function after long-term TAC. We hypothesized, therefore, that ATF6β controlled a similar subset of genes whose expression is activated by UPR signaling in the heart like ATF6α. To examine the targets controlled by these 2 transcription factors in the heart we created doxycycline (DOX)-repressible cardiac-specific transgenic mice expressing the transcriptionally-active N-termini of either ATF6α (dubbed ATF6α constitutively-nuclear, ATF6α-cn), or ATF6β (similarly dubbed ATF6β-cn) (Fig. 3a). This system requires accessory tTA protein expression associated with a second cardiac-specific transgene that binds the tetracycline operator (TetO) in the promoter of the first transgene. Constitutive ATF6α-cn expressing mice (no DOX) were normal as young adults by echocardiography and gravimetric analysis; however ATF6β-cn mice did not survive to weaning. To circumvent this issue, ATF6α-cn and ATF6β-cn mice were raised on DOX food to repress expression of the transgene until adulthood. At 8 weeks of age (4 weeks after removal of DOX) each line was shown to have increased expression of *Atf6* or *Atf6b* message (Table S2) and when examined by echocardiographic and gravimetric analysis, each was found to be overtly normal (data not shown). Hearts from these mice were subjected to global gene array analysis using the Clariom S microarray platform. Because expression levels of ATF6α-cn and ATF6β-cn were not equal, a direct comparison between the two transcription factors was not possible (and probably would still not be possible even if levels were equal, due to differences in protein stability^13,14^). However, it was clear that a subset of genes upregulated at least 2-fold by ATF6α-cn or ATF6β-cn fell into overlapping sets of gene ontology (GO) categories (Fig. 3b). From these shared data groups we extracted many of the upregulated genes, which included a variety of ER protein chaperones, ER stress-associated genes, and genes involved in ERAD, and confirmed their upregulation via qPCR (Table S2) or by immunoblotting for ER stress proteins such as Uggt1, PERK, Hyou1, IRE1, Heat shock protein 90b1 (Hsp90b1), BiP, calreticulin, Herpud1, elongation initiation factor 2α (eIF2α) or ARMET from ATF6α-cn and ATF6β-cn transgenic ventricular tissue (Fig. 3c and quantified in Fig. S5).

**Figure 4:**
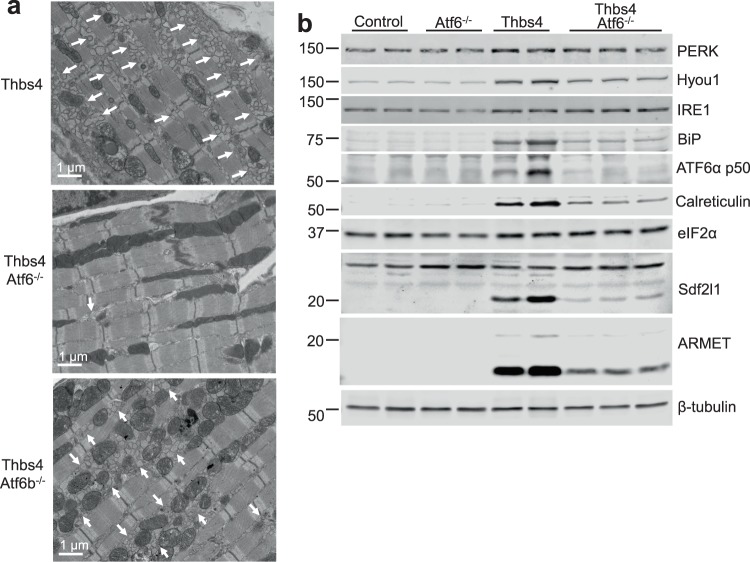
Thbs4-mediated ER expansion and upregulation of ER stress-associated proteins requires ATF6α. (**a**) Transmission electron micrograph of sections from Thbs4 DTG, Thbs4 DTG and *Atf6*^−/−^, or Thbs4 DTG and *Atf6b*^−/−^ hearts. Arrows mark areas of increased ER and the post ER-vesicular compartment. (**b**) Representative immunoblots for the indicated ER stress-associated proteins from heart homogenates of control, *Atf6*^−/−^, Thbs4, or Thbs4 *Atf6*^−/−^ mice.

The observation that ATF6β functioned very similar to ATF6α in the heart as an adaptive ER stress regulator during hypertrophy suggested that each transcription factor might function similarly downstream of Thbs4, although our previous observations suggested Thbs4 only activated ATF6α^2^. To examine this concept we crossed *Atf6*^−/−^ or *Atf6b*^−/−^ mice with mice harboring the Thbs4 and tTA transgenes that we previously generated (Figs 1a,b,e and S1). Electron microscopy revealed massive expansion of the ER and vesicular compartments in Thbs4 and Thbs4 *Atf6b*^−/−^ mice, while all Thbs4-induced ER expansion was lost in the *Atf6*^−/−^ background (Fig. 4a). Similarly, immunoblotting experiments found that upregulation of the previously-described panel of ER stress associated proteins by Thbs4 required the expression of ATF6α (Fig. 4b and quantified in Fig. S6). These results indicate that ATF6α uniquely regulates ER compartment expansion downstream of Thbs4, while ATF6β was not involved in Thbs4-dependent regulation in the heart.

## Discussion

Hypoxia due to ischemic injury after MI injury alters the oxidative environment of the ER and impairs disulfide bond formation^16^, while the increase in protein production accompanying hypertrophic growth of the heart can dramatically increase the load of nascent proteins that ER chaperones must contend with^3,17^. In addition, cardiac disease produces alterations in myocyte Ca^2+^ handling, aided by upregulation of proteins like STIM1, which is a Ca^2+^ sensor involved in ER Ca^2+^ loading in non-excitable cells that we have shown underlies cardiac pathology^18^. In response to cardiac disease states the ER signals through three primary stress response pathways: PERK, IRE1, and ATF6α^4^. In particular, ATF6α has been the focus of several recent studies due to the largely protective gene program it appears to induce after acute MI or I/R injury^2,7–9^.

We previously demonstrated that activation of ATF6α by its C-terminal binding partner Thbs4 is sufficient to induce a protective ER stress response that also resulted in expansion of the ER and downstream vesicular compartments, along with upregulation of ER stress-associated proteins^2,19^. Subsequent work in cultured cells revealed that Thbs4-mediated expansion of the ER and vesicular compartments required ATF6α, and that adenoviral expression of ATF6α-cn was sufficient to induce vesicular expansion in neonatal cardiac myocytes^20^, which is consistent with our findings presented here (Fig. 4a). However, despite the evidence that Thbs4-mediated ATF6α activation is protective after acute injury, we did not observe that overexpression of Thbs4 mitigated phenotypic changes associated with cardiac hypertrophy (Fig. S1). Instead, we observed that loss of ATF6α or ATF6β initially reduced cardiac hypertrophy but in the long term it accelerated cardiac decompensation and failure after pressure overload.

Importantly, *Atf6b*^−/−^ gene-deleted mice appeared to show the same defects in cardiac hypertrophy, mobilization of ER protein chaperones, and ultimately underwent the same accelerated failure with pressure overload as their *Atf6*^−/−^ counterparts (Fig. 2a–c). These observations were surprising because ATF6β was previously described as a weak transcription factor that functioned as an inhibitor of ATF6α in the heart^13–15^. Our data suggest that ATF6β serves a role similar to ATF6α in the heart by upregulating a partially-overlapping set of genes also involved in preservation of ER function and ERAD activity (Fig. 3b,c, Table S2), in agreement with results in INS-1 cells^21^. This observation is also consistent with the fact that *Atf6*^−/−^ *Aft6b*^−/−^ (double nulls) are embryonic lethal, as are 3 of 4 allele-deleted mice (in either direction, data not shown). However, we speculate that the longer half-life of ATF6β^13,14^ coupled with its relatively weaker transcriptional activity^13–15^ results in UPR signaling that is functionally similar to the more labile ATF6α protein. While it seems clear from our results that both ATF6α and ATF6β regulate a shared subset of genes that we believe is important for pressure overload compensation, we have not at this time identified which of these genes play the most important roles in cardioprotection (though this is a focus of our ongoing research), and this is admittedly a limitation of the current study.

While ATF6β shares a basic pattern of processing with ATF6α that includes disinhibition by loss of BiP binding, trafficking to the Golgi, and sequential proteolytic processing that releases the transcriptionally-active N-terminus^22^, ATF6β does not require Thbs4 for the initial trafficking step, as we have previously demonstrated for ATF6α^2^. Indeed, the effects of Thbs4 do not appear to require ATF6β whatsoever (Fig. 4a). While the N-termini including the transactivation and DNA binding bZIP domains of ATF6α and ATF6β have high identity, it was the divergent c-terminus in ATF6α that uniquely interacted with Thbs4^2^. Thus, we would predict that ATF6β engages with unique regulatory partners that may provide a pattern of regulation distinct to that of ATF6α under select stress conditions, and identification of these partner proteins is currently ongoing.

Finally, while our data strongly suggest that both ATF6α and ATF6β are important for the development of compensatory cardiac hypertrophy following pressure overload stress, it is unclear whether the major activities of these proteins are confined to myocytes, or if they also play important roles in other cell types in the heart such as cardiac fibroblasts, endothelial cells, and immune cells. Thus, the role of ATF6α and ATF6β in these non-myocyte cells in regulating cardiac homeostasis and disease responsiveness is a focus of our ongoing research.

## Methods

### Gene-deleted and transgenic mice

Gene-deleted mice lacking ATF6α or ATF6β protein were obtained from the lab of Kazutoshi Mori (Kyoto University, Kyoto, Japan) and were previously described^10^. Thbs4 transgenic mice were previously described^2^. To create ATF6α-cn and ATF6β-cn transgenic mice a cDNA encoding mouse ATF6α^1–364^ fused N-terminal to a FLAG tag or mouse ATF6β^1–388^ fused N-terminal to a Myc tag were cloned into the modified murine α-myosin heavy chain promoter expression vector that is tetracycline operator dependent^23^ and used to inject newly-fertilized oocytes (*C57BL/6* background). These responder transgenes were bred with transgenic mice expressing the tetracycline transactivator (tTA) protein that is driven by the murine α-myosin heavy chain promoter, and in the absence of tetracycline or doxycycline (DOX) expression is induced, but with DOX administration expression is suppressed. Because ATF6β-cn double transgenic mice exhibited early lethality, we administered DOX in the chow to repress transgene expression until weaning. For experiments in Figs 1a,b,e, 4a–b and S1, mice were in a mixed *FVB/N* and *C57BL/6* background. For all other experiments, mice are in a pure *C57BL/6* background. For pressure overload experiments in Figs 1 and 2, TAC surgeries were performed on mice at 8–10 weeks of age. For experiments in Fig. 3, transgenic mice were 8 weeks of age (4 weeks off DOX chow). Both sexes of mice were used and no animals were discarded in the statistical analysis. For Fig. 1a,b, the tTA sham group contained 2 female and 2 male mice, the tTA TAC group contained 11 female and 4 male mice, the *Atf6*^−/−^ tTA sham group contained 3 female and 3 male mice, the *Atf6*^−/−^ tTA TAC group contained 10 female and 9 male mice, the *Atf6b*^−/−^ tTA sham group contained 2 female and 2 male mice, and the *Atf6b*^−/−^ tTA TAC group contained 4 female and 8 male mice. For Fig. 1c,d, the Wt sham group contained 13 female and 9 male mice, the Wt TAC group contained 16 female and 3 male mice, the *Atf6*^−/−^ sham group contained 3 female mice and 1 male mouse, the *Atf6*^−/−^ TAC group contained 5 female and 6 male mice, the *Atf6b*^−/−^ sham group contained 4 female mice, and the *Atf6b*^−/−^ TAC group contained 13 female mice, 11 male mice, and 4 mice of unrecorded sex. For Fig. 2a,c, the Wt control group contained 5 female and 7 male mice, the Wt TAC group contained 4 female and 20 male mice, the *Atf6*^−/−^ control group contained 7 female and 2 male mice, the *Atf6*^−/−^ TAC group contained 10 female and 10 male mice, the *Atf6b*^−/−^ control group contained 2 female and 3 male mice, the *Atf6b*^−/−^ TAC group contained 8 female and 4 male mice, the *Atf6*^+/−^ *Atf6b*^+/−^ control group contained 2 female and 4 male mice, and the *Atf6*^+/−^ *Atf6b*^+/−^ TAC group contained 2 female and 9 male mice. For Fig. 2b, group distribution is the same as in Fig. 2a,c, except that the *Atf6*^−/−^ TAC group contains 11 female and 10 male mice, and the *Atf6b*^−/−^ control group contains 2 female mice and 1 male mouse. For Fig. S1, the tTA expressing groups have the same sex distribution as described for Fig. 1a,b. For Thbs4 and tTA transgene expressing groups, the Wt sham group contained 2 female and 2 male mice, the Wt TAC group contained 9 female and 3 male mice, the *Atf6*^−/−^ sham group contained 4 female and 3 male mice, the *Atf6*^−/−^ TAC group contained 11 female and 10 male mice, the *Atf6b*^−/−^ sham group contained 3 female and 2 male mice, and the *Atf6b*^−/−^ TAC group contained 8 female and 6 male mice. In order to confirm that there were no sex differences within our experimental groups, we performed one-way ANOVA with Newman-Keuls post-hoc test on groups from Figs 1a–d, 2a–c, and S1a–d in which there were at least 3 male and 3 female data points and found no significant differences between female and male mice within each group. Experiments involving animals were approved by the Institutional Animal Care and Use Committee of Cincinnati Children’s and in accordance with the National Institutes of Health Guidelines for the care and use of laboratory animals.

### Echocardiography and pressure overload induction

Mice were anesthetized with 2% isofluorane by inhalation. Echocardiography was performed in M-mode using a Hewlett Packard (Palo Alto, CA, USA) SONOS 5500 instrument with a 15 MHz transducer. For pressure overload induction, mouse littermates aged 8–10 weeks were subjected to transverse aortic constriction (TAC) or a sham surgical procedure, as previously described^24^. For TAC experiments in Fig. 1, controls for each genotype are sham-operated mice. For TAC experiments in Fig. 2, controls for each genotype are sham-operated and non-surgical mice of the corresponding age. Doppler echocardiography was performed on mice subjected to TAC in order to determine pressure gradients across the aortic constriction. Surgeries and echocardiography were conducted in a blinded manner.

### Western blotting

Hearts were excised from mice, frozen in liquid nitrogen, and stored at −80 °C. Ventricles were homogenized in a buffer containing 20 mM Tris-HCl, pH 7.5, 250 mM NaCl, 1% Triton X-100, 0.5 mM dithiothreitol, and protease inhibitors. Homogenates were centrifuged at 14,000 rpm for 10 min and supernatants were used for immunoblotting. Protein extracts were subjected to SDS-PAGE and transferred to PVDF membranes. Immunoblots were performed using the appropriate primary antibody and fluorescent conjugated secondary antibodies (LI-COR, Lincoln, NE, USA) in combination with an Odyssey CLx Infrared Imaging System (LI-COR). Primary antibodies used were: PERK (Cell Signaling Technology, Danvers, MA, USA), Hyou1 (Lifespan, Providence, RI, USA), IRE1 (Cell Signaling Technology), BiP (Sigma-Aldrich, St. Louis, MO, USA), PDI (Cell Signaling Technology), calreticulin (Cell Signaling Technology), ARMET (Abcam, Cambridge, UK), Sdf2l1 (Sigma-Aldrich), β-tubulin (Developmental Studies Hybridoma Bank, Iowa City, IA, USA), Uggt1 (Santa Cruz Biotechnology, Dallas, TX, USA), Hsp90b1 (Sigma-Aldrich), Herpud1 (Cell Signaling Technology), Eif2α (Cell Signaling Technology), and ATF6α (Signalway Antibody, College Park, MD, USA). Quantification of immunoblots was performed using Image Studio software (LI-COR, Lincoln, NE, USA) and normalized to β-tubulin in all cases. The values indicated in Fig. S3 are set relative to that for the average of Wt TAC samples on the same gel. The values indicated in Figs S5–6 are set relative to that for the average of control samples on the same gel. Uncropped gel files with molecular weight markers of immunoblots are shown in Figs S7–S10.

### Gene array and mRNA analysis

Microarray analysis was carried out using the Affymetrix Clariom S platform (Thermo Fisher Scientific, Waltham, MA, USA) at the Gene Expression Core Facility at Cincinnati Children’s Hospital Medical Center (Cincinnati, OH, USA). Bioinformatic interrogation of data (CEL files) was performed to determine differential gene expression with the Transcriptome Analysis Console (Applied Biosystems, Foster City, CA, USA; ver. 4.0.0.25), the Clariom_S_Mouse TAC Configuration file (ver. 1), and the iPathwayGuide (Advaita Bioinformatics, Plymouth, MI, USA). Validation of select microarray results was achieved by quantitative PCR (qPCR); briefly, RNA was extracted from ventricles using the RNeasy Kit according to manufacturer’s instructions (Qiagen, Hilden, DEU) and cDNA was synthesized by reverse transcribing total RNA using oligo-dT primers and Superscript III First-strand synthesis Kit (Life Technologies, Carlsbad, CA, USA). Real-time PCR was performed using SsoAdvanced SYBR Green (Bio-Rad Technologies, Hercules, CA, USA), with Gapdh as the reference gene for normalization. Fold-change in expression levels was calculated using the delta-delta-CT method. Statistical significance of differences between groups was assessed by t-test analysis of raw replicate dCT values.

### Histology and electron microscopy

Analysis of myocyte hypertrophy was performed in a blinded fashion by measuring the minimum Feret’s diameter from haemotoxylin and eosin (H&E) stained paraffin sections^12^. Collagen content was measured via Picrosirus Red staining (Electron Microscopy Sciences, Hatfield, PA, USA) of paraffin sections from the indicated number of hearts. Images were evaluated with NIH Image J software to determine percent collagen versus total myocyte area. TUNEL staining (Roche, Indianapolis, IN, USA) was also conducted after antigen retrieval on paraffin heart histological sections according to the kit protocol. Immediately after, sections were stained with cardiac troponin T antibody reactivity (Abcam, Cambridge, UK) and DAPI to enrich the percentage of TUNEL nuclei from cardiomyocytes. Electron microscopy was performed on heart sections from Thbs4, Thbs4 *Atf6*^−/−^, or Thbs4 *Atf6b*^−/−^ mice as previously described^2^.

### Statistics

Results are presented in all cases as mean ± SEM. Statistical analysis was performed using Prism 7 (Graphpad Software, La Jolla, CA, USA). Experiments were analyzed using one-way ANOVA with Newman-Keuls multiple comparisons test, or for qPCR experiments, unpaired Student’s t-test. P-values less than 0.05 were considered significant.

## Supplementary information

Supplemenatary figures and tables

## Data Availability

Gene expression study data are deposited to NCBI Gene Expression Omnibus (GEO) database (#GSE124797) and will be released after the embargo period. All other data for this study are contained within the paper as primary figures, supplementary figures, or supplementary tables.
