# Supplementary material for: Overlapping and differential functions of ATF6α versus ATF6β in the mouse heart
Source: Sci Rep. 2019 Feb 14;9:2059. doi: 10.1038/s41598-019-39515-5 (PMC6375966; doi:10.1038/s41598-019-39515-5)
Supplement: Supplementary file 1 — Supplemenatary figures and tables [file 41598_2019_39515_MOESM1_ESM.pdf]

# Overlapping and differential functions of ATF6α versus ATF6β in the mouse heart

**Robert N. Correll[1,2], Kelly M. Grimes[2], Vikram Prasad[2], Jeffrey M. Lynch[2], Hadi Khalil[2], Jeffery D. Molkentin[2,3]***

[1] Department of Biological Sciences, University of Alabama, Tuscaloosa, AL, 35487 USA
[2] Department of Pediatrics, University of Cincinnati, Cincinnati Children's Hospital Medical Center, Cincinnati, Ohio, 45229 USA
[3] Howard Hughes Medical Institute, Cincinnati, Ohio, 45229 USA
[*] Corresponding author: jeff.molkentin@cchmc.org

## Supplementary Figures and Tables

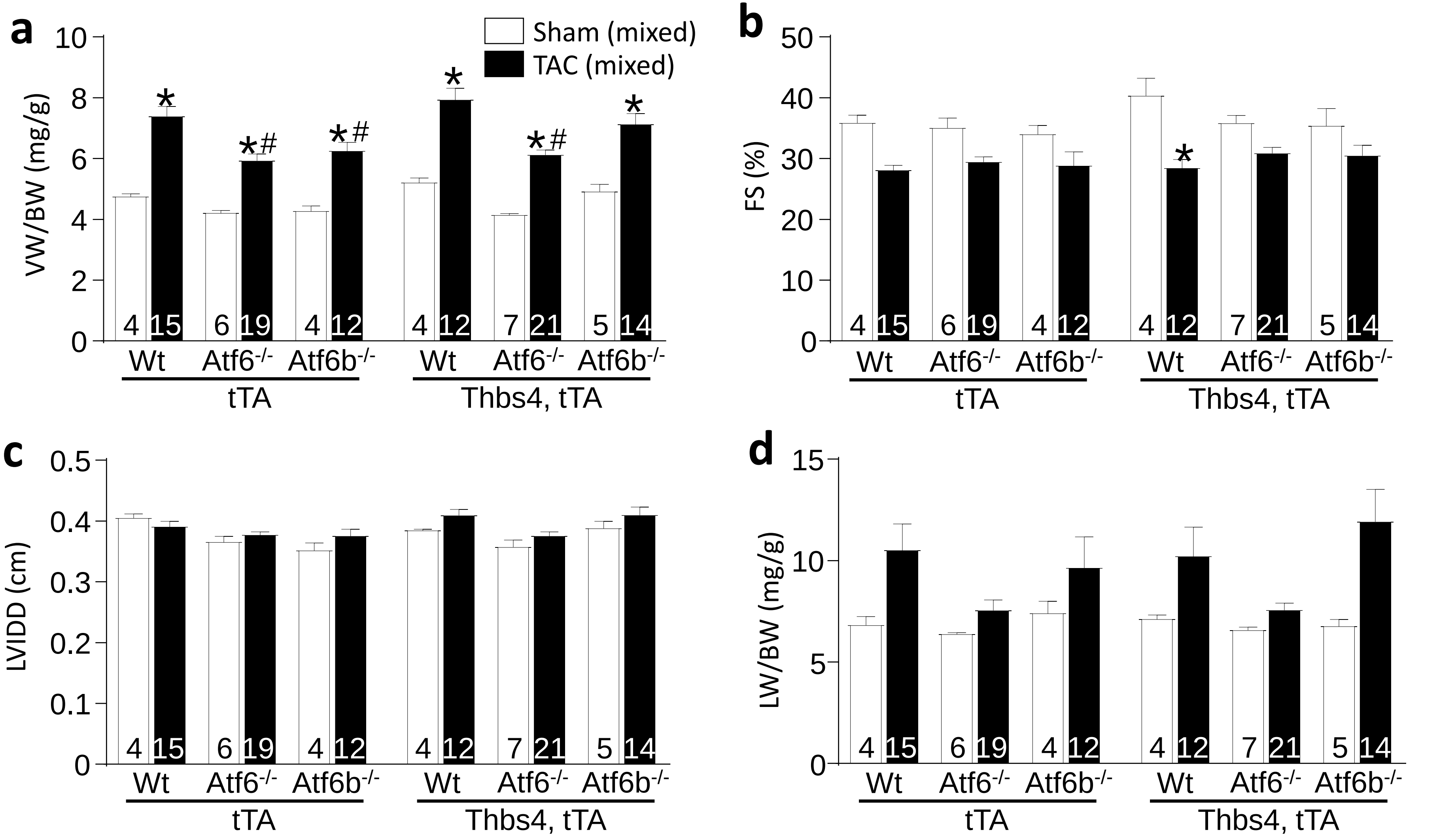


**Figure S1. Assessment of cardiac hypertrophy and structure function in mice lacking ATF6α or ATF6β protein with or without the Thbs4 transgenes.** (**a**), Gravimetric measurement of ventricle weight normalized to body weight (VW/BW), (**b**), echocardiographic measurement of fractional shortening percentage (FS%), (**c**), echocardiographic measurement of left ventricular interior diastolic dimension (LVIDD), and (**d**), gravimetric measurements of lung weight normalized to body weight (LW/BW) from tTA *Atf6*$^{-/-}$, tTA *Atf6b*$^{-/-}$, Thbs4 *Atf6*$^{-/-}$, Thbs4 *Atf6b*$^{-/-}$, and tTA or Thbs4 control mice after 2 weeks of TAC or a sham surgery. *P<0.05 versus sham of same genotype; #P<0.05 vs tTA TAC (for tTA hearts) or Thbs4 TAC, which also have the tTA transgene (Newman-Keuls multiple comparisons test).

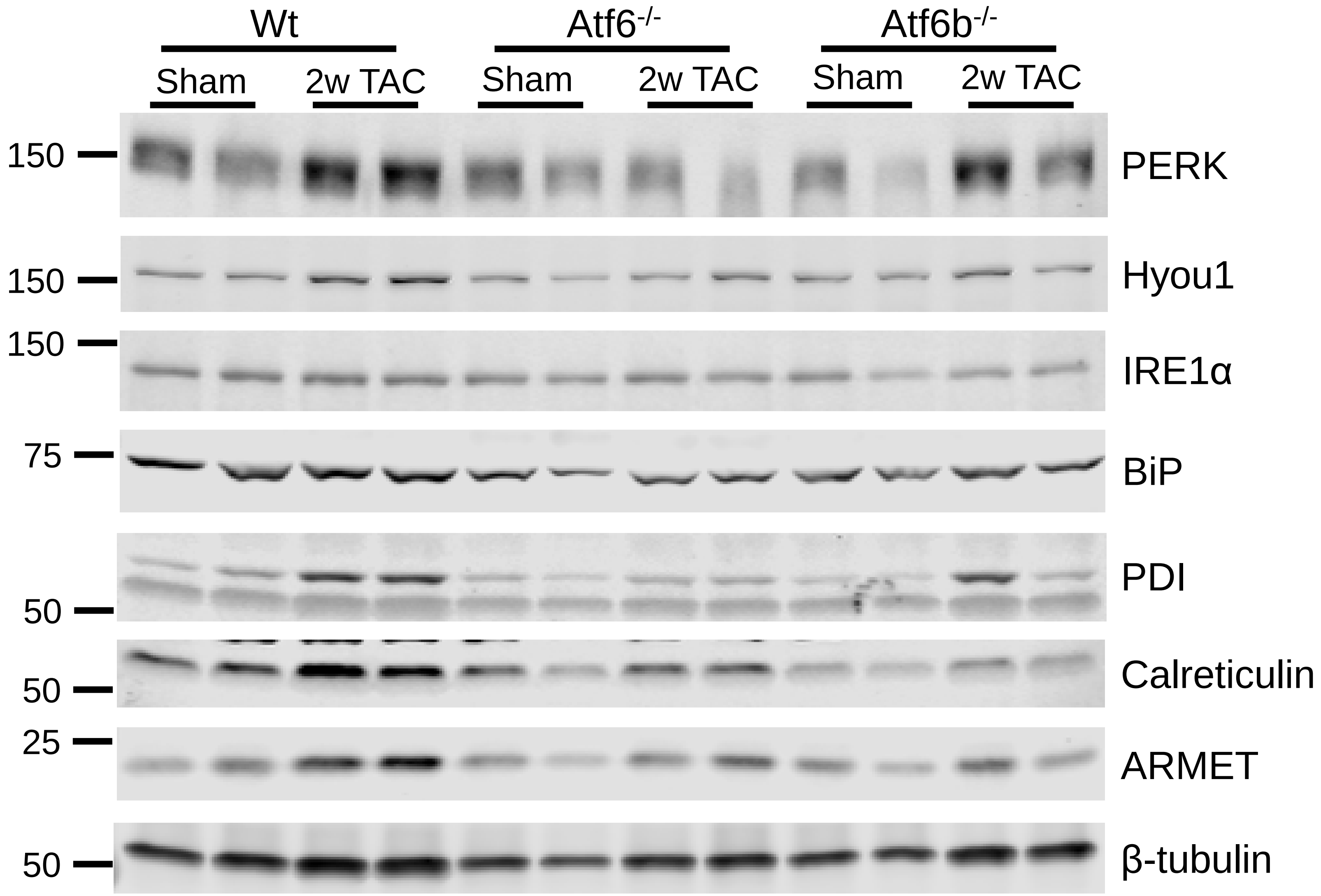


**Figure S2. Immunoblots of ER stress responsive proteins after 2 weeks of TAC.** Lysates were derived from control or 2w TAC operated Wt, *Atf6*$^{-/-}$, or *Atf6b*$^{-/-}$ mice in a pure *C57BL/6* background (experiment described in Fig. 1c,d).

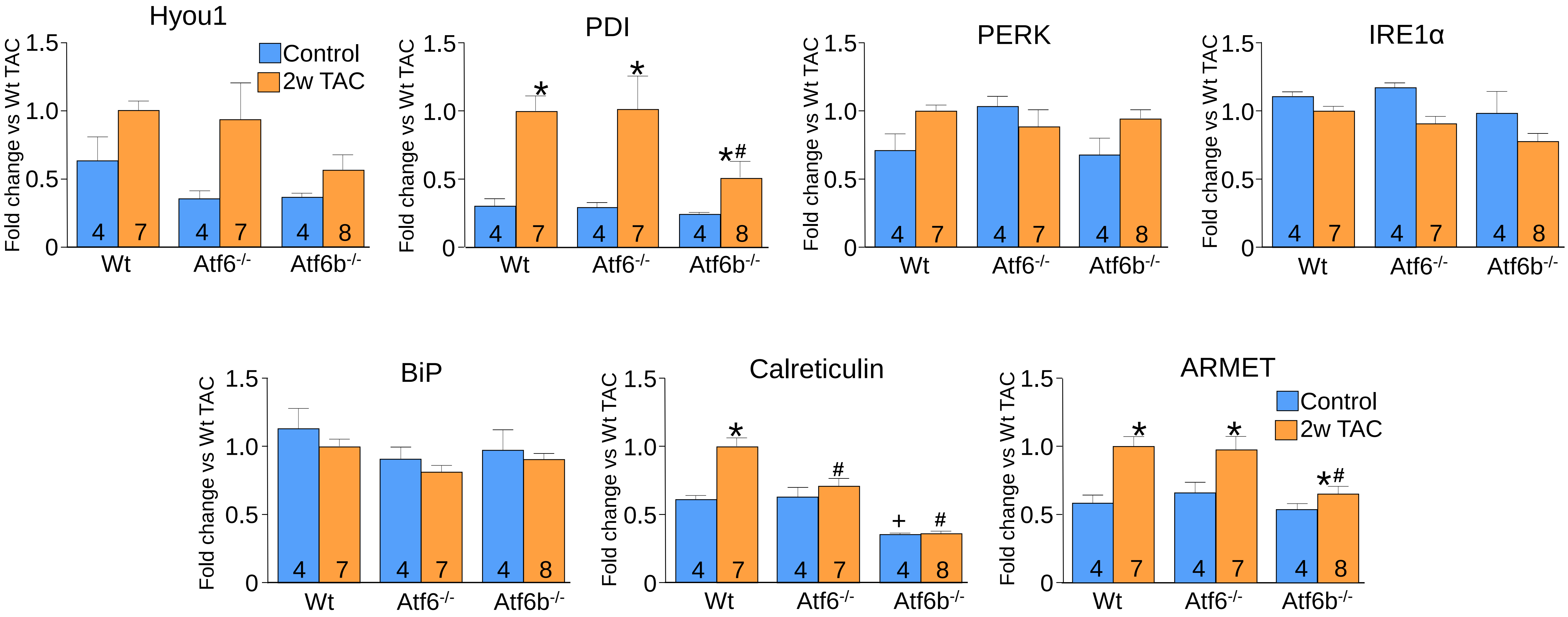


**Figure S3. Quantification of immunoblots of ER stress-responsive proteins after 2 weeks of TAC.** Quantification of immunoblots depicted in Fig. S2 and other blots derived from the same pool of samples (not shown). Values for each sample were normalized to β-tubulin and set relative to the value for Wt TAC on each individual blot. For each experiment, number of mice analyzed is given within the graph. *P<0.05 versus control of same genotype; #P<0.05 vs Wt TAC for TAC comparisons only; +P<0.05 vs Wt Sham for sham vs sham comparisons only; (Newman-Keuls multiple comparisons test).

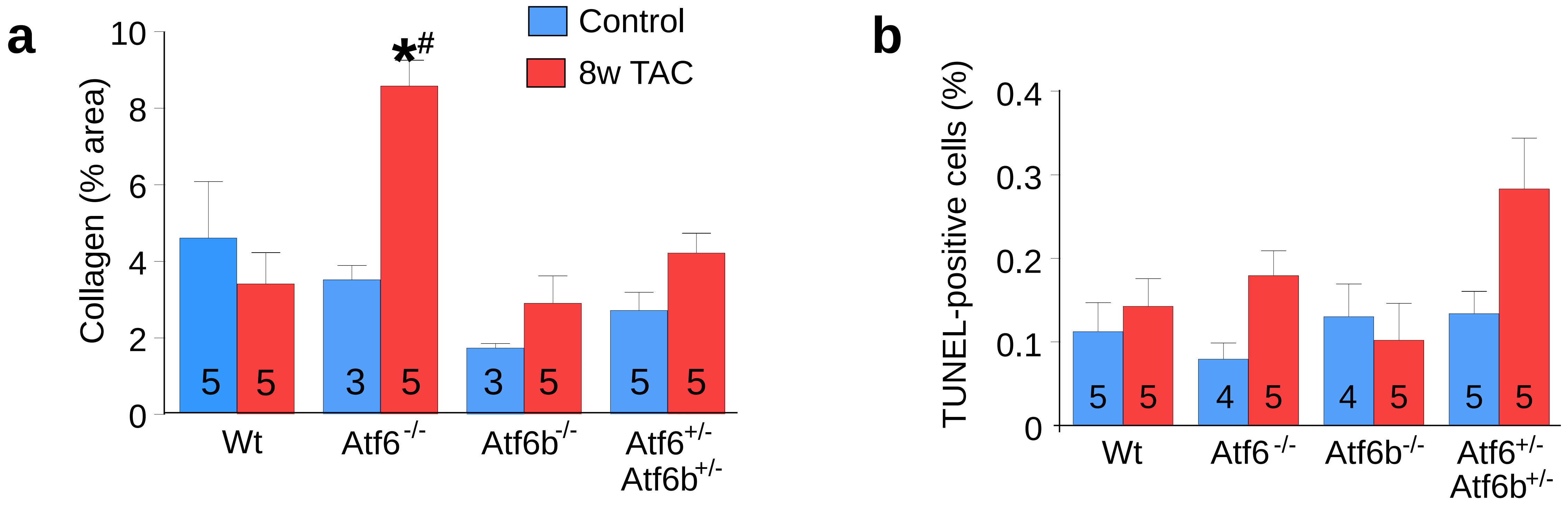


**Figure S4. Analysis of collagen content and TUNEL after long-term TAC.** (a) Quantification of collagen-containing area from Picrosirus red stained heart histological sections, and (b) quantification of TUNEL-positive nuclei from cardiac histological sections taken from *Atf6*$^{-/-}$, *Atf6b*$^{-/-}$, *Atf6*$^{+/-}$ *Atf6b*$^{+/-}$, or control mice after 8 weeks of TAC or a sham surgery. Sections were also stained for cardiac troponin T to enrich for scoring cardiomyocytes. Number of mice analysed is shown in the bars of each panel. *$P<0.05$ versus sham of same genotype; #$P<0.05$ vs Wt TAC for TAC comparisons only (Newman-Keuls multiple comparisons test).

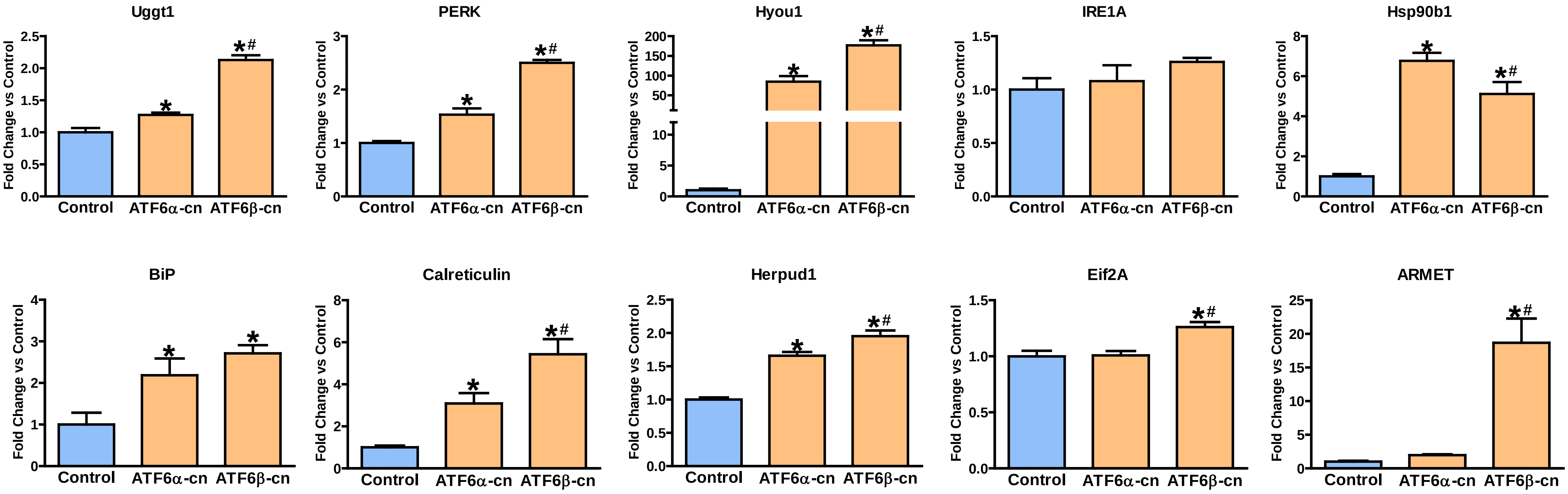


**Figure S5. Quantification of immunoblots from Figure 3c.** All three samples were analyzed for each treatment. Values for each sample were normalized to β-tubulin and set relative to the control value on each individual blot. *P<0.05 versus control; #P<0.05 vs ATF6α-cn for ATF6β-cn comparisons only (Newman-Keuls multiple comparisons test).

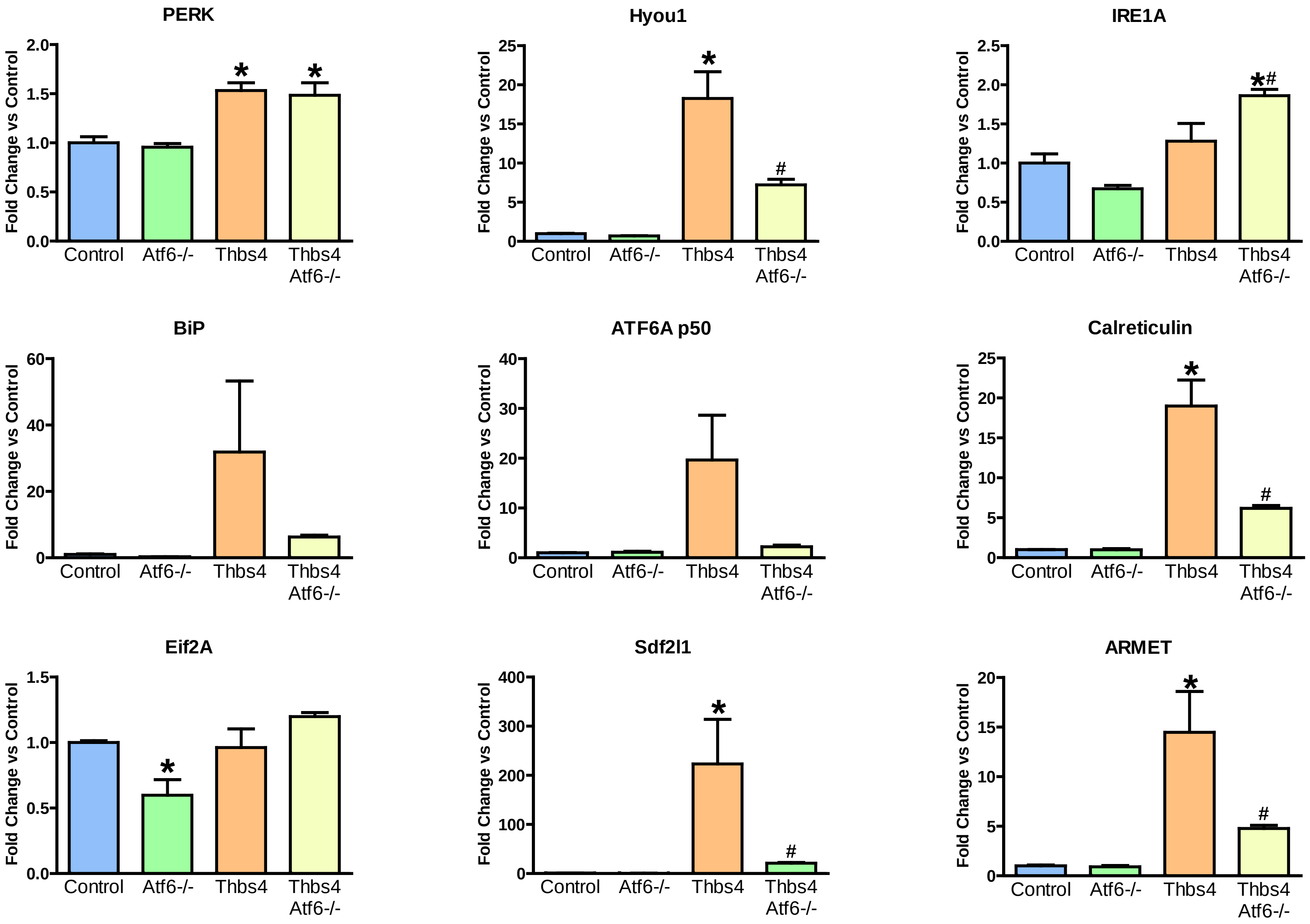


**Figure S6. Quantification of immunoblots from Figure 4b.** Three samples analyzed for each treatment. Values for each sample were normalized to β-tubulin and set relative to the control value on each individual blot. *P<0.05 versus control; #P<0.05 vs Thbs4 for Thbs4, *Atf6*$^{-/-}$ comparisons only (Newman-Keuls multiple comparisons test).

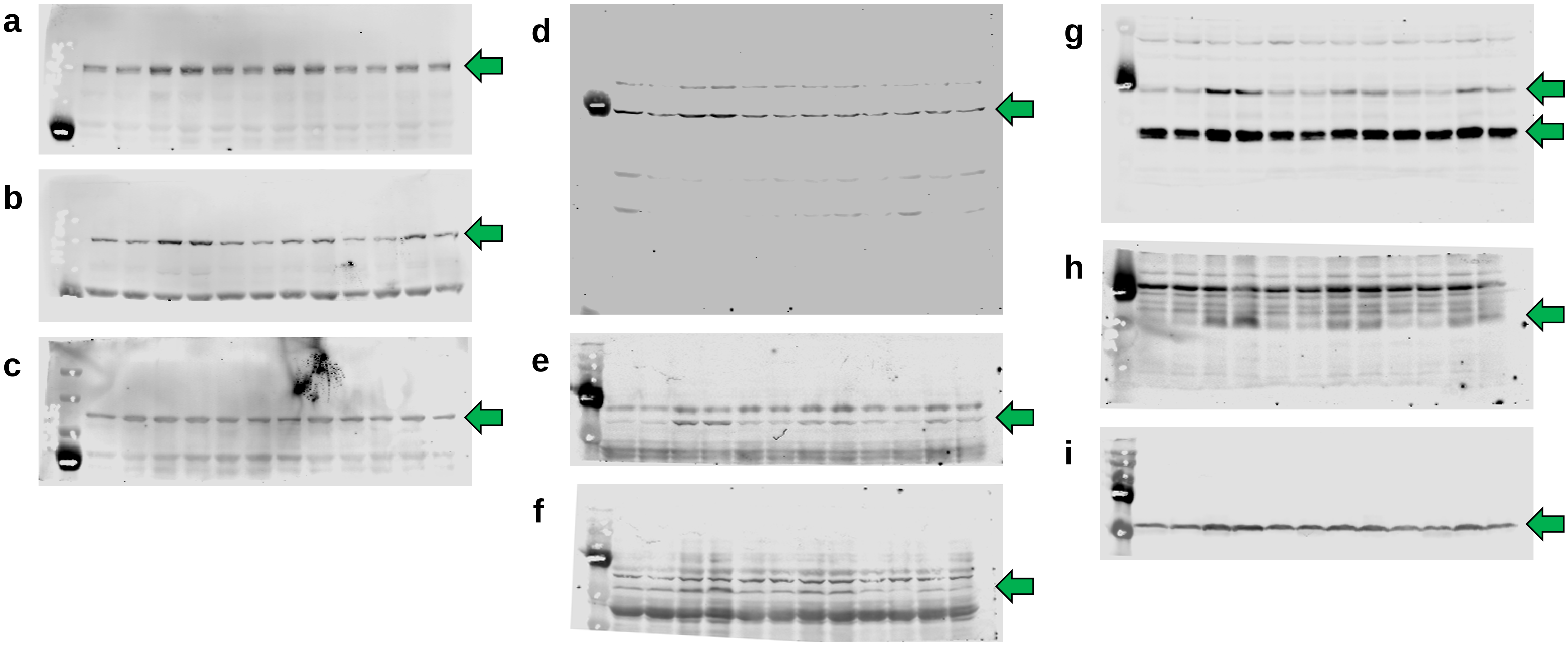


**Figure S7. Uncropped images of immunoblots from Figure 1.** Immunoblots of (**a**) PERK, (**b**) Hyou1, (**c**) IRE1α, (**d**) BiP, (**e**) PDI, (**f**) calreticulin, (**g**) ARMET, (**h**) Sdf2l1, and (**i**) β-tubulin from Fig. 1e. In all cases bands of interest are marked with a green arrow. In some cases, membranes were cut prior to immunoblotting. Panels **S7a-c**, **S7e-f, and S7i** represent the top part of the membrane. Panels **S7g-h** represent the bottom part of the membrane.

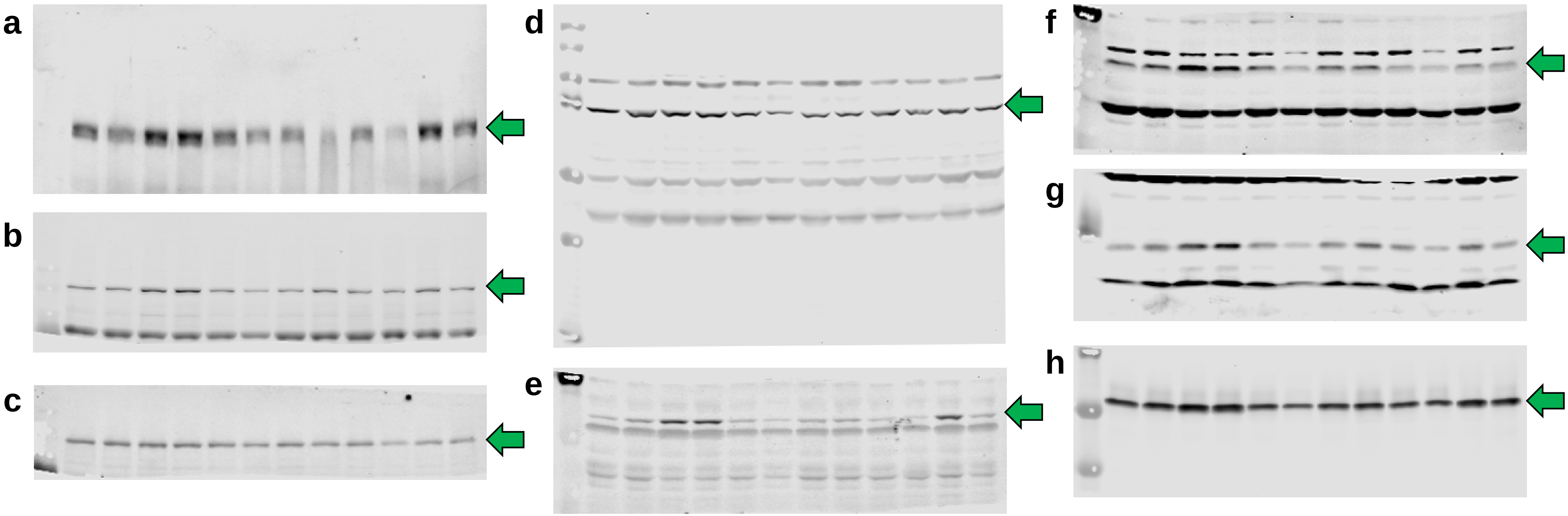


**Figure S8. Uncropped images of immunoblots from Figure S2.** Immunoblots of (**a**) PERK, (**b**) Hyou1, (**c**) IRE1α, (**d**) BiP, (**e**) PDI, (**f**) calreticulin, (**g**) ARMET, and (**h**) β-tubulin from Fig. S2. In all cases bands of interest are marked with a green arrow. In some cases, membranes were cut prior to immunoblotting. Panels **S8a-c** represent the top part of the membrane. Panels **S8e-h** represent the bottom part of the membrane.

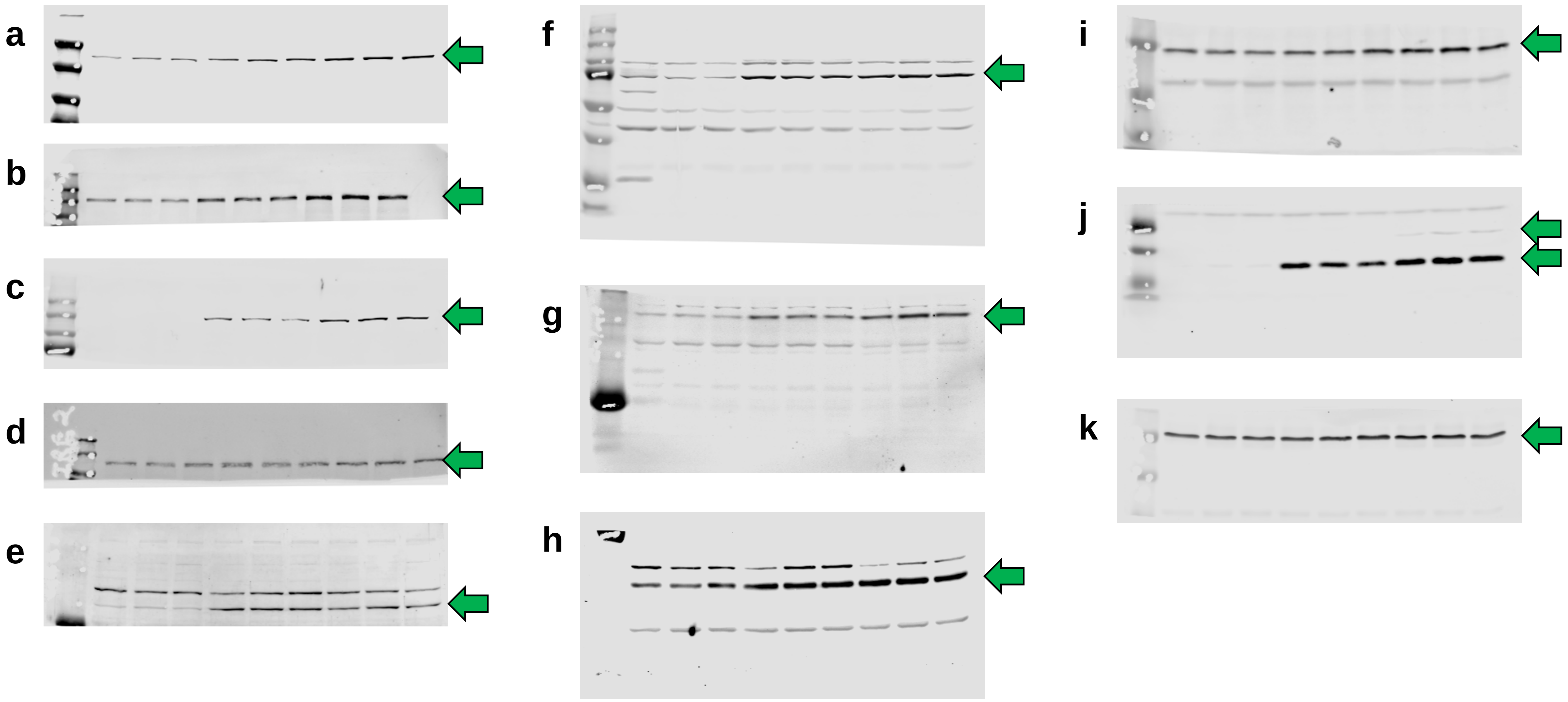


**Figure S9. Uncropped images of immunoblots from Figure 3.** Immunoblots of (**a**) Uggt1, (**b**) PERK, (**c**) Hyou1, (**d**) IRE1, (**e**) Hsp90b1, (**f**) BiP, (**g**) calreticulin, (**h**) Herpud1, (**i**) Eif2α, (**j**) ARMET, and (**k**) β-tubulin from Fig. 3c. In all cases bands of interest are marked with a green arrow. In some cases, membranes were cut prior to immunoblotting. Panels **S9a-e** represent the top part of the membrane. Panels **S9g-k** represent the bottom part of the membrane.

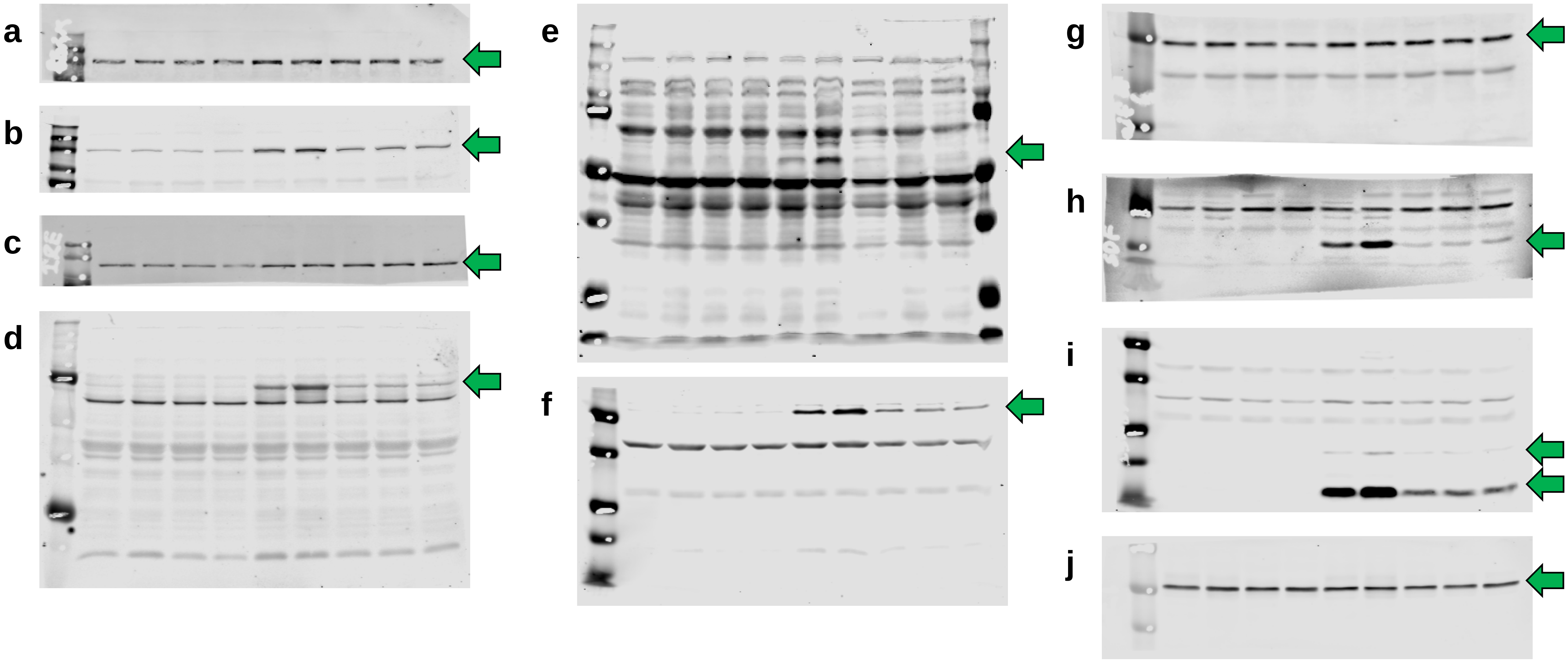


**Figure S10. Uncropped images of immunoblots from Figure 4.** Immunoblots of (**a**) PERK, (**b**) Hyou1, (**c**) IRE1, (**d**) BiP, (**e**) ATF6α p50, (**f**) calreticulin, (**g**) Eif2α, (**h**) Sdf2l1, (**i**) ARMET, and (**j**) β-tubulin from Fig. 4b. In all cases bands of interest are marked with a green arrow. In some cases, membranes were cut prior to immunoblotting. Panels **S10a-c** represent the top part of the membrane. Panels **S10f-j** represent the bottom part of the membrane.

**Fold change (TAC vs Sham)**

| | Genes Tested | | | |
|---|---|---|---|---|
| **Genotype** | **ANF** | **BNP** | **Myh6** | **Myh7** |
| **WT** | 12 | 2.33 | 0.48 | 4.7 |
| **Atf6-/-** | 13.4 | 4.39 | 0.82 | 5.18 |
| **Atf6b-/-** | 5.32 | 5.57 | 0.7 | 180.1 |

**Table S1. Hypertrophic marker gene analysis.** mRNA fold change of atrial natriuretic factor (ANF), brain natriuretic peptide (BNP), α-myosin heavy chain (Myh6) and β-myosin heavy chain (Myh7) measured via qPCR from control, *Atf6*$^{-/-}$, or *Atf6b*$^{-/-}$ hearts after 2 weeks of TAC vs a sham surgery. All values are relative to sham, which was set to 1 for each mRNA analyzed.

| | Fold Upregulation | | |
|---|---|---|---|
| **Gene** | **ATF6α TG** | **ATF6β TG** | **Function** |
| **Atf6** | **3.51** | **0.0891** | ATF6 arm of UPR |
| **Atf6b** | **0.836** | **29.1** | ATF6 arm of UPR |
| **Derl3** | **1450** | **210** | ERAD component |
| **Herpud1** | **2.17** | **3.39** | ERAD component |
| **Hsp90b1** | **4.62** | **2.67** | Chaperone, ERAD component |
| **Syvn1** | **2.98** | **2.78** | E3 ligase, ERAD component |
| **Dnajb9** | **2.2** | **5.63** | Chaperone, ERAD component |
| **Fkbp7** | **2.42** | **6.15** | PPIase, chaperone |
| **Sel1l** | **4.66** | **3.46** | ERAD component |
| **Uggt1** | **5.68** | **7.99** | Glucosyltransferase, ER quality control |
| **Xbp1** | **2.37** | **2.33** | IRE1 arm of UPR |

**Table S2. mRNA levels from hearts of ATF6α-cn versus ATF6β-cn.** mRNA fold change from hearts of selected genes taken from shared microarray GO categories from 8 week-old (4 weeks off DOX) ATF6α-cn or ATF6β-cn transgenic mice vs. Wt control hearts.
